# Telemedicine: the experience of health professionals in the supplementary sector

**DOI:** 10.1590/1980-220X-REEUSP-2022-0374en

**Published:** 2023-03-31

**Authors:** Heloísa Pimenta Arruda Araújo, Lucas Cardoso dos Santos, Rúbia Aguiar Alencar

**Affiliations:** 1 Universidade Estadual Paulista “Júlio de Mesquita Filho”, Faculdade de Medicina de Botucatu, Departamento de Enfermagem, Botucatu, SP, Brazil.; 2 Universidade de São Paulo, Escola de Enfermagem, São Paulo, SP, Brazil.

**Keywords:** Telemedicine, Supplemental Health, Qualitative Research, Telemedicina, Salud Complementaria, Investigación Cualitativa, Telemedicina, Saúde Suplementar, Pesquisa Qualitativa

## Abstract

**Objective:**

To know the experience of health care professionals about telemedicine in a supplementary health service.

**Method:**

This is a qualitative study carried out in a health clinic in the city of São Paulo with 12 participants selected through intentional sampling. Data collection took place through semi-structured interviews and data processing followed the methodological framework of Bardin content analysis.

**Results:**

The speeches that emerged addressed telemedicine in the context of professional training, the care modalities carried out through digital technologies, the benefits and challenges identified in the practice of telemedicine for work and care processes.

**Conclusion:**

The need for public policies and training was identified, aimed at improving understanding of digital health, its modalities and potential in care, teaching and research environments and in health organizations, aligned with training for the use of telemedicine as the one that qualifies the care provided by health professionals.

## RESUMO

**Objetivo:**

Conhecer a experiência de profissionais de saúde acerca da telessaúde em um serviço de saúde suplementar.

**Método:**

Pesquisa de natureza qualitativa realizada em um ambulatório de saúde no município de São Paulo com 12 participantes selecionados por meio de amostragem intencional. A coleta de dados ocorreu por meio de entrevistas semiestruturadas e o processamento dos dados seguiu o referencial metodológico da análise de conteúdo de Bardin.

**Resultados:**

Os discursos emergidos abordaram a telessaúde no contexto de formação profissional, as modalidades de cuidado realizadas por meio de tecnologias digitais, os benefícios e desafios identificados na prática da telessaúde para os processos de trabalho e de cuidar.

**Conclusão:**

Identificou-se a necessidade de políticas públicas e de formação voltadas a melhorar a compreensão quanto à saúde digital, suas modalidades e potencialidades nos ambientes assistenciais, de ensino e pesquisa e das organizações de saúde, alinhadas à formação para o uso da telessaúde como aquela que qualifica o cuidado ofertado pelos profissionais de saúde.

## RESUMEN

**Objetivo:**

Conocer la experiencia de los profesionales sanitarios sobre la telesalud en un servicio complementario de salud.

**Método:**

Investigación cualitativa realizada en un ambulatorio de la ciudad de São Paulo con 12 participantes seleccionados por muestreo intencional. La recopilación de datos se realizó mediante entrevistas semiestructuradas y el tratamiento de los datos siguió el marco metodológico del análisis de contenido de Bardin.

**Resultados:**

Los discursos emergentes abordaron la telesalud en el contexto de la formación profesional, las modalidades de atención realizadas mediante tecnologías digitales, los beneficios y desafíos identificados en la práctica de la telesalud para los procesos de trabajo y de atención.

**Conclusión:**

Se identificó la necesidad de políticas públicas y de formación destinadas a mejorar la comprensión sobre la salud digital, sus modalidades y potencialidades en entornos asistenciales, en entornos de docencia e investigación y en las organizaciones sanitarias, alineadas con la formación para el uso de la telesalud como herramienta que cualifica la atención ofrecida por los profesionales de la salud.

## INTRODUCTION

Faced with advances in information technology and transformations in human communication, the intersection between the areas of health and technology is presented as a strategy for facing the challenges permeating health systems. This fusion of the areas of technology and health configures what is called digital health^(1)^.

The term digital health has different definitions that converge on a common objective: to promote the safe use of Information and Communication Technologies (ICT) to contribute to the improvement of the individuals’ quality of life and citizenship. The insertion of digital technologies in health has been widespread, with several positive impacts on care, on the work process, and on teaching and research scenarios. Among these benefits, professional qualification, flexibility in the care model, feasibility of access to services, and interoperability between systems are highlighted, which together have the potential to improve management and quality of care^(1)^.

In 2015, the United Nations recognized the need to significantly increase access to ICTs as one of the means to achieve the 17 sustainable development goals^(2)^. Later, in 2019, the World Health Organization (WHO) prepared the Global Strategy on Digital Health guideline, unifying under the term “digital health” all the concepts of application of ICT in health, defining it as “the field of knowledge and practice associated with the development and use of digital technologies to improve health”, from inception to operation^(3, p.5)^.

In this movement, and in the midst of the diversity of terms related to digital health, the WHO also defined the term telemedicine as the use of ICT for the provision of services by health professionals aimed at allowing the exchange of information for the purpose of diagnosis, disease treatment and prevention, including research, evaluations, and continuing education^(4)^. Other nomenclatures are used with the prefix tele-, in which its definition will be linked to the radical used, such as: teleconsultation, teleconsultancy, tele-education, telemonitoring, and teleguidance.

The coronavirus pandemic (*corona virus disease* - COVID-19) boosted telemedicine in Brazil and in the world, making it a common and necessary practice, in view of the circulation restrictions due to the transmissibility of the virus and the need for agility in the organization of services^(5,6)^.

At an international level, there is an increase in telemedicine, with a 10% growth from 2020 to 2021 and 43% of adults who reported using telemedicine services during the pandemic with a preference to maintain this practice^(5)^. On the national scene, this context is portrayed by a study that points to an increase of almost 77% in teleconsultations when comparing to the same period of 2019 with 2020^(6)^.

In Brazil, the use of ICT is guaranteed by Organic Law No. 8.080 (1990), when considering the incorporation of scientific and technological development. In 1991, with the creation of the Department of Informatics of the Brazilian Public Health System (SUS), information systems were created. Since then, some milestones in digital health in the country have brought about changes in health services, such as the General Law for the Protection of Personal Data, the National Policy on Information and Informatics, and the National Policy on Technological Innovation in Health^(7)^.

More recently, Ordinance No. 1.434, of May 28, 2020, established rules for the use of ICTs, establishing the *Connect SUS* Program and amending Consolidation Ordinance No. 1/GM/MS, of September 28, 2017, to institute the National Health Data Network, which seeks to promote the exchange of information in the Health Care Network, allowing the transition and continuity of patient care involving the public and private sectors and presenting legal sustainability in the General Law for the Protection of Personal Data^(8)^.

The National Health Data Network allows the interoperability of health information among different health establishments and management agencies, as it has already occurred with the *Conecte-SUS* portal, used since the beginning of the pandemic by professionals in public and private services and by the citizens themselves^(7)^.

However, there are barriers to the progress of digital health, such as the allocation of financial resources, the lack of training at the undergraduate and graduate levels in health focused on the use of ICTs, the difficulties related to digital literacy, incipient continuing education actions in the services, and obstacles regarding access to technologies and connection networks^(4)^.

Therefore, the exponential growth of telemedicine as a resource for the care and the gap in the literature regarding the experience of health professionals in digital care in the supplementary sector show that it is necessary to know the experience of health professionals about telemedicine, aiming at the possibilities, advances and challenges, to bring reflections and contributions to training, professional performance, and health care.

When taking the experience of health professionals from the supplementary sector as the object of this study, we recognized it as an important factor in the qualification of digital health, strengthening this practice in what regards increasing the capacity for care provision, facilitating access to qualified care, reducing service overload, and helping to organize flows in the health system^(9)^.

In view of the above, the study aims to know the experience of health professionals about telemedicine in a supplementary health service, assuming as a question of this study: how do health professionals in the supplementary sector experience telemedicine as a resource for care?

## METHODS

### Design of Study

This was a qualitative study^(10)^ guided by the theoretical framework of telemedicine^(4)^ that sought to know the experience of the actors involved in the use of ICTs in health and the value attributed to them as a strategy for the care of patients in a supplementary health service, taking the steps proposed by the *Consolidated Criteria for Reporting Qualitative Research* (COREQ) into account^(11)^.

### Local

The present study was carried out in a health clinic of a large philanthropic institution in the city of São Paulo, with approximately 16,795 patients registered at the time of the study and with consultations taking place from Monday to Friday, from 7:00 a.m. to 8:00 p.m., individually or in a group, in-person or remotely, also being possible to take place through consultations shared among professionals from different categories.

In the pre-COVID-19 pandemic period, in-person consultations were the majority; however, as of March 2020, there has been an inversion in this logic of care in which telemedicine care has grown exponentially, especially teleconsultations and telemonitoring of symptomatic respiratory patients.

### Population and Selection Criteria

The outpatient clinic has 35 professionals, nine administrative assistants and 26 health professionals: four family nurses, nine family and community physicians, two dieticians, two psychologists, and nine nursing technicians. An intentional sample was used for the selection of participants as a way for the researcher to select a group of the population that was representative of the whole^(12)^. Inclusion criteria were: being a health professional at the selected clinic, having worked in telemedicine for at least three months between March 2020 and July 2021 in the organization, and being interested in collaborating with this research. The exclusion criteria were being on vacation or sick leave.

### Sample Definition

Of the 26 health professionals, two were on vacation, seven were nursing technicians who did not include the use of digital technologies in their scope of work, and the other two professionals had been at the institution for less than 30 days. Therefore, 15 professionals were invited to participate in this study: four nurses, five family physicians, two nutritionists, two psychologists and two nursing technicians. Of this total, 12 accepted the invitation and three declined.

### Data Collection

Data collection took place through semi-structured, audio- recorded interviews, with a duration of 25 to 50 minutes and an average of 36 minutes, and use of a guided script to capture the experience of telemedicine professionals between June 29 and July 22, 2021, by a female researcher, with an undergraduate course in nursing and a graduate course in family health.

Considering that no instruments were found to assess the professional’s experience with telemedicine, we opted for the elaboration of a specific script, based on the research question and grounded on the theoretical framework of telemedicine, which was reviewed by other researchers with expertise in qualitative research and submitted to a pre-test/pilot phase, with no changes occurring after this phase.

The final instrument consisted of 18 open questions, with the first five questions related to demographic, academic, and occupational characterization, followed by those focused on living with telemedicine, individual or shared professional performance through telemedicine, identification of training carried out by the professional to work through telemedicine, knowledge regarding ethical, legal, and digital safety aspects, perceptions about telemedicine as a care modality, the patient’s perception from the professional’s point of view, and possible previous experiences of professionals as a patient from a digital service.

The audios of the interviews were transcribed in full and then sent by email to the participants for them to read, adding comments and/or even improving the answers, but no feedback was received.

### Data Analysis and Treatment

The processing of the collected data followed the methodological framework of content analysis proposed by Bardin, which is considered a set of techniques with the use of systematic and objective steps to describe different discourses and highlight the nature and strength of the stimuli to which the participant is subjected^(13)^.

**Chart 1.**
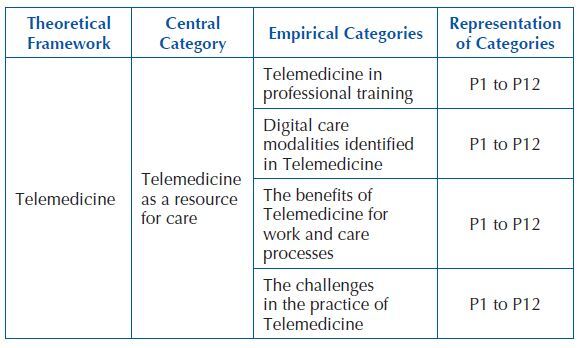
Thematic table with presentation of the theoretical framework, central category, empirical categories, and categories representation, from the interviews, São Paulo, SP, Brazil, 2022.

Source: Data collected through the self-authored instrument.

At first, through the collected information, the participants were characterized and, subsequently, the reports were individually analyzed and named with the letter P - due to the condition of being a participant - followed by the interview number. Material organization and thorough reading allowed the systematization of the initial ideas of the study, with the central category and empirical categories of the study (Chart 1) being then defined.

### Ethical Aspects

The present study complied with Resolution no. 466/2012 of the National Health Council, with the Free Informed Consent Form being applied prior to the interviews, and was approved by the Research Ethics Committees of the proponent and co-participant Institutions with number of opinions 4.668.451 (04/24/2021) and 4.778.288 (06/14/2021), respectively.

## RESULTS

Data were structured into four empirical categories (Chart 1) shown below, with 12 participants being part of the study, most of them female (75%), aged between 29 and 38 years old, mean age 33 years old, coming from technical courses in nursing and undergraduate courses of nursing, medicine, nutrition, psychology, with an average of ten years of practice. Working time at the institution in the sector under investigation ranged between four and 36 months, with an average of 26 months.

### Telemedicine in Professional Training

The category is about the context, the learning opportunities about telemedicine, the aspects of telemedicine training before the COVID-19 pandemic and the initiatives provided by the analyzed institution that represented the trigger for this practice in the participants’ daily lives.

In the field of professional training, most participants brought experiences with digital technologies that occurred at different times throughout undergraduate and graduate courses, but in a punctual and infrequent way, causing them not to recognize digital health in their practice.

*We already had online courses and lectures, but it wasn’t that often, it was something new, innovative, you know? (P6)*

In the wake of health training involving ICT, professionals reported that during the undergraduate course they perceived resistance and prejudices in relation to telemedicine and that digital health was something futuristic, distant, informal and with risks for care provision with no quality and safety.

*On graduation, it was something unimaginable and even condemned (…) it was like an affront, like, ‘how does the person want me to prescribe something without seeing them in person?! (P10)*

A large part of the participants claimed that the transition from exclusively in-person care to mixed care was a sudden and challenging change in the midst of the COVID-19 pandemic, which led to the need for training and qualification on digital health.

To this end, permanent education actions aimed at digital health were present even though they were not focused on the use of digital technologies and their different modalities as a way to enhance and amplify care, being directed to the technical use of platforms and systems, the preparation and setting for remote assistance.

*When I started, yes, they taught about the platforms, how to access, how the flow would be (…) How to talk, how to approach, how to conduct. (P4)*

*(In the midst of the COVID-19 Pandemic), it was not valid, we need to provide care. We had no preparation for this (…). We learned what worked. (P11)*

Contradictions are observed in the speeches regarding having or not guidelines for the use of ICT and it is evident in the reports that training and continuing education actions were proposed based on the needs identified by the management. The sudden implementation of telemedicine as a result of the COVID-19 pandemic still appeared as a justification for digital care to occur even without adequate training.

### Digital Care Modalities Identified in Telemedicine

The second category addresses the different ways professionals use to exchange information through technological tools with the aim of composing health care. The digital care modalities remembered by the participants were tele-education, tele-consultation and tele-guidance associated, respectively, with the continuing education activities proposed by the institution, consultations carried out at an individual and collective level, and guidance focused on health care.

*(…) she wasn’t going to be able to bring her daughter to change the dressings, we made a video call, she advised (…) then we took a picture, even sent it to the orthopedist. (P6)*

*(…) health education is something we do a lot (…) education, prevention, general, educational guidelines, transmission of information. (P7)*

In view of the moment of the COVID-19 pandemic, telemonitoring, usually carried out by telephone, gained space and strength to monitor the progression of respiratory symptoms and provide relevant guidance to patients who could not be exposed to the health environment or those who remained in isolation at home.

Other telemedicine practices used by the teams for holding team meetings, shared consultations and case discussions were also mentioned, which not only allowed interaction with professionals who were working remotely, but also strengthened and qualified care and teamwork.

### The Benefits of Telemedicine for Work and Care Processes

This category dialogues with the gains perceived by the participants in carrying out digital care, portraying this practice as a potentiality for access to services and professionals, to qualify the health care offered and for the acquisition of individual and collective skills that contribute to the professional development.

The participants listed the potentialities in the use of Telemedicine in its different aspects, with digital health being the most prominent one as an enhancer of care.

*(…) Previously, the patient would only know if the exam was altered in the consultation (…) I think this was a very important gain from telemedicine. (…) Another thing is the faster follow-up appointments. (P5)*

*(…) due to telemedicine, it seems that we are in the patient’s home (…) he shares much more with us than it would be in an office. (P8)*

Digital health presents itself as a way to facilitate access to professionals, allowing required care to be directed and provided in different situations, in addition to providing assistance that is closer to the reality of each user.

Participants also reported that the benefits of telemedicine brought positive impacts to professional practice that allowed and contributed to the development of skills for the use of ICTs, such as better use of digital resources for coordination and continuity of care, better professional-professional and professional-patient interaction, and better adherence to meetings and training.

Moreover, the use of digital platforms appeared as a way to facilitate patients’ access to health services and of professionals to patients, reducing distances, avoiding unnecessary displacements and exposures inherent to health services.

*(…) we manage to reach places that we would not reach and it reduces time too, you know? This optimizes our time. (P8)*

It is noticed that the participants recognized telemedicine as a promising change, bringing the desire for it to further improve health services and systems.

### The Challenges of Telemedicine Practice

Contrasting the potentialities identified and mentioned above, the fourth category called lists the obstacles related to telehealth that were identified by the participants in their practice.

Among the challenges reported, the most representative was the lack of physical contact and, sometimes, isolated verbal communication, without the video, which ended up limiting, in the perception of some, the emotional support and the process of understanding the user and being understood.

*(…) when it’s on the phone, we can’t see the person’s face, you know, this is lacking. To see how the person is… Sometimes, through video, you can identify this and end up questioning a few more things. (P3)*

It appears then that the lack of visual resource, through audio calls, and physical contact with the patient were listed as limitations for care, as well as communication that appeared as a point of greater attention in the sense of validating information and making oneself understood.

Another difficulty pointed out by the participants was the connection and mastery of digital platforms and tools by users, making this process more challenging and sometimes hindering the provision of care.

*(…) we find many people who do not know what Zoom, Teams are, much less open them there, save an application on their cell phone. (P3)*

Such obstacles related to the connection and management in the use of digital tools were presented as a consensus among the participants and that, in most cases, were linked to patients, especially in advanced age groups or those who did not have access to digital devices and Internet connection.

Finally, there was a unanimous perception that updates are needed in relation to the available digital tools, especially regarding the need to have interoperable systems that make up the care process, whether in person or digitally.

*An integrated system, I think. That you could assist the patient via video, open the medical record, send him the information you had in that consultation. Referrals, prescriptions, everything. (P5)*

Telemedicine was seen by professionals as a promising and permanent practice and that is why there is a need for investments not only in the interoperability of systems, but also in professional training for this type of care, enhancing its benefits and overcoming its weaknesses.

## DISCUSSION

The present study allowed knowing the experiences of health professionals with telemedicine, showing that it is configured as a more frequent practice in the work environment than in undergraduate and graduate training settings.

In the Brazilian educational scenario, it is clear that, in the period before the pandemic, the teaching structure in undergraduate and graduate courses was mostly in-person and telemedicine appeared at specific times as an alternative tool for training, remote classes and courses, reducing costs and facilitating the development of permanent education strategies aimed at health professionals and not specifically as a modality for care^(14)^.

Faced with the pandemic context caused by COVID-19 and the need to recognize telemedicine as a care tool for services, educational institutions have reformulated their methods by inserting audiovisual materials, video classes, virtual therapeutic groups and teleconsultations^(15,16)^.

These changes in teaching methods were made possible and driven by Ordinance No. 343 of March 17, 2020, by allowing the replacement of in-person classes with classes taught by digital means as part of the Ministry of Education’s action plan with the objective of maintaining the routine of studies and avoiding losses in teaching during the pandemic^(17)^.

The use of technologies in teaching methods, called digital education, has the potential to make the learning process more dynamic and flexible, providing educators with different methodologies that, if well selected, can expand student knowledge and provide opportunities for familiarization with ICTs for the practice of digital health^(15,18)^.

Dialoguing with the results of this study, initiatives presenting telemedicine as a tool that composes care and prepares future professionals for the practice are extremely important^(19,20)^. Therefore, there is a need to provide students with simulated practices in a controlled teaching environment so that they are better prepared for professional practice.

In the field of professional practice, the Brazilian legislation of professional councils on Digital Health has changed with the COVID-19 pandemic. Law No. 13.979, of February 6, 2020, defined the measures to face the COVID-19, regulated and operationalized through Ordinance No. 467, of March 20, 2020, which, on an exceptional and temporary basis, authorized Telemedicine actions between the medical professional and the patient in pre-clinical care, care support, consultation, monitoring and diagnosis, through ICTs, within the scope of the SUS, and supplementary and private health^(21,22)^.

Faced with the sanitary context, the federal and regional councils of other health professional categories mobilized themselves to create resolutions that would guide the application of ICTs in professional practice, as was the case of the Federal Council of Nursing, among other federal councils of several health professional categories^(23)^.

Study participants reported training activities that sought to qualify and update professionals on their practice with telemedicine, but in a non-standardized way and focused on the needs identified by the management of the service. This fact raises reflections on the pertinence of actions aligned with the theoretical framework of permanent education based on the problematization of the work process and with the objective of qualifying health professionals to transform the practice^(24,25)^.

In the field of professional practice, studies show that, in addition to teleconsultations, telemedicine has frequently been presented through tele-education, telemonitoring and teleconsultations actions, to enhance professional performance, teamwork, and the care provided^(9,20,26)^, data that dialogue with the findings of this research, especially in the telemonitoring of symptomatic respiratory patients.

Telemedicine presents itself as a possibility to facilitate access and communication among professionals, patients and services, allowing better care planning based on team meetings and case discussions with the use of different tools and digital resources, as demonstrated by the participants of this study^(27)^.

Contrasting the benefits presented in the use of telehmedicine for educational, work and care processes, it is necessary to recognize the challenges involving its implementation, such as: understanding telemedicine and its different modalities; ensuring the necessary infrastructure for connectivity and interoperable systems; professional training to provide quality remote assistance; and the implementation of protocols and institutional guidelines that guide this practice in a safe way^(26)^.

The adaptation of professionals and users to the use of telemedicine is the most cited obstacle, also made explicit in the participants’ speeches. Resistance and difficulty in adapting to the new model of care on the part of professionals is related to the lack of theoretical and practical knowledge on the subject, a fact that is directly related to the need for investments in training and continuing education activities, and with the lack of mastery reported by the participants about the theoretical framework of telemedicine^(28,29)^.

In this regard, an American study emphasized that, in the face of the pandemic, professionals and users needed to adapt to communication through digital platforms, with changes in teaching curricula being required, to provide professionals with the opportunity to develop new skills that qualify virtual communication^(28)^.

The challenges perceived regarding the users are related to the lack of necessary skills or infrastructure, such as access to devices and internet connection, which is very much related to the lack of digital literacy^(28,29)^.

A cultural barrier is especially present in the elderly population or those with less technical knowledge and ability to use digital platforms. Aiming to overcome these barriers, the adoption of mechanisms that guarantee the inclusion of specific populations is recommended and discussion is required on the relationship between subjects and technologies and on digital literacy being a competence developed to optimize and allow the effective implementation of telemedicine actions^(27)^.

Thus, the importance of discussing the role of digital technologies in care with different social actors, such as health professionals, users, teachers and managers, is evident. Therefore, it is necessary that digital literacy, understood as the set of skills necessary for the use of ICT, can guide the educational processes to form subjects prepared to autonomously adhere to rapid technological changes^(30)^.

Emphasizing this statement, existing evidence reinforces health literacy as an essential competence for professionals to ensure effective communication and person-centered care, leading to a reduction in inequalities and improvement in outcomes. In this context, nurses are identified as important health education agents, showing that health literacy teaching cannot be neglected^(31)^.

This way, it is worth reflecting that for the implementation of telemedicine, the various variables involved in this process shall be recognized. That is, it is necessary to combine preparation and skills for handling digital tools with financial resources, willingness to try this new practice, access to services that offer this type of care, connectivity and adequate infrastructure.

In view of the results obtained, it is expected that this research can bring implications for the practice of services and contribute to the discussion of the practice of telemedicine as the one that qualifies the care provided by health professionals, and reinforce the importance and need for initiatives from the training institutions and health organizations aimed at training professionals to use telemedicine.

The limitations of this study stem from the fact that the research was carried out in a pandemic moment, which ends up showing a cutout of a particularly specific moment.

## CONCLUSIONS

The study allowed getting to know the experience of health professionals from a supplementary sector service regarding telemedicine, as well as their experiences in the context of training. In this regard, the importance of public policies and training is highlighted, to improve understanding of digital health and that its possibilities in practice are stimulated, considering the exponential growth in the use of ICT in care, teaching and research environments, and its power to qualify care and expand access to services.

It also reinforces the need to promote updates to health professionals to qualify and enhance their practice through ICT, especially initiatives promoting understanding and debate about digital health for professional qualification and care. Finally, the importance of research that considers telemedicine in the context of the supplementary sector is highlighted.
